# Clock at the Core of Cancer Development

**DOI:** 10.3390/biology10020150

**Published:** 2021-02-14

**Authors:** Sonal A. Patel, Roman V. Kondratov

**Affiliations:** 1 Fusion Pharmaceuticals Inc., Hamilton, ON L8P 0A6, Canada; patel@fusionpharma.com; 2 Department of Biological, Geological and Environmental Sciences, Cleveland State University, Cleveland, OH 44115, USA

**Keywords:** circadian clock, core clock genes, cancer, metabolism, chemotherapy, immunotherapy, radiation

## Abstract

**Simple Summary:**

The earth’s rotation produces a daily 24 h cycle of day and night. Many biological functions, such as sleep–wake cycle, feeding/fasting cycle, blood hormone levels and body temperature rhythms occur over a 24 h period. These daily fluctuations are controlled by an internal system known as the circadian clock (Latin circa—“around”—and diem—“day”). This clock coordinates the periodic changes in diverse physiological and behavioral activities and helps adapt to the periodic environment for the benefit of survival and reproduction of organisms. Nearly all organisms, from single-cell to mammals, including humans, possess a self-sustained circadian clock. Disturbances in the functioning of this internal circadian clock can alter the balance in biological functions and compromise organism fitness. In humans, clock disruption is associated with diseases such as diabetes, cardiovascular diseases and cancer. Thus, studying the interplay between the circadian clock and physiology is important in the prevention and management of fatal diseases such as cancer.

**Abstract:**

To synchronize various biological processes with the day and night cycle, most organisms have developed circadian clocks. This evolutionarily conserved system is important in the temporal regulation of behavior, physiology and metabolism. Multiple pathological changes associated with circadian disruption support the importance of the clocks in mammals. Emerging links have revealed interplay between circadian clocks and signaling networks in cancer. Understanding the cross-talk between the circadian clock and tumorigenesis is imperative for its prevention, management and development of effective treatment options. In this review, we summarize the role of the circadian clock in regulation of one important metabolic pathway, insulin/IGF1/PI3K/mTOR signaling, and how dysregulation of this metabolic pathway could lead to uncontrolled cancer cell proliferation and growth. Targeting the circadian clock and rhythms either with recently discovered pharmaceutical agents or through environmental cues is a new direction in cancer chronotherapy. Combining the circadian approach with traditional methods, such as radiation, chemotherapy or the recently developed, immunotherapy, may improve tumor response, while simultaneously minimizing the adverse effects commonly associated with cancer therapies.

## 1. Introduction

**Figure 1:**
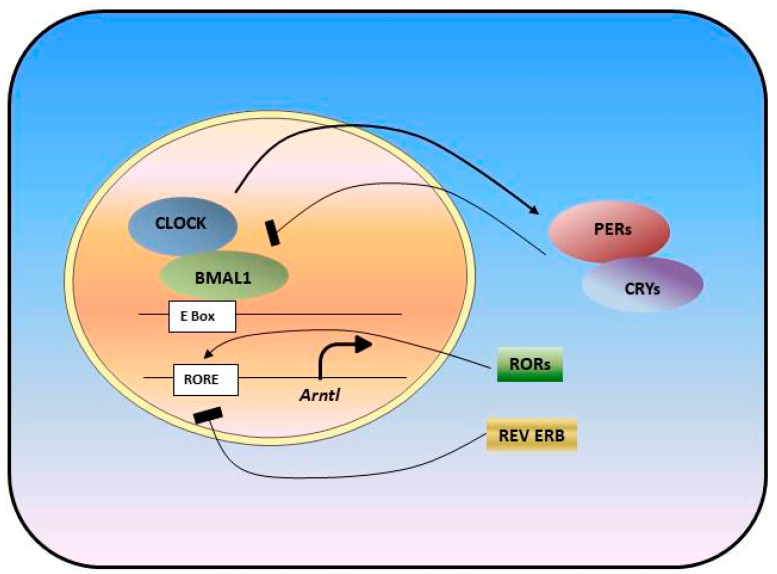
Molecular Circadian Oscillator is formed as a transcriptional translational feedback loop. Transcription factors, BMAL1 and CLOCK, drive the transcription of clock genes: *Pers, Crys, Rev-Erbs, Rors.* PER and CRY proteins, in turn, heterodimerize and inhibit CLOCK/BMAL1 complex activity and their own transcription, which form one loop. Rev-Erbs and RORs form a second loop and act by either repressing or activating *Bmal1* transcription.

The circadian clock is a time-keeping mechanism that controls daily rhythms in biological processes [1,2,3]. The environmental light/dark cycle entrains the master clock in the suprachiasmatic nucleus (SCN) [4,5]. The central clock, in turn, sends neural and humoral signals to synchronize the peripheral clocks in different organs such as liver, kidney, skin, intestine, lung, pancreas, ovary, and heart to maintain the energy balance and homeostasis in our body. The feeding/fasting cycle can also serve as a clue for peripheral clocks [6]. At the molecular level, circadian clocks consist of an intricate network of transcription–translation feedback loops formed by dozens of genes and their products, of which expressions and activities fluctuate rhythmically across the day [7,8]. Transcription factors circadian locomotor output cycles kaput (CLOCK) and ARNTL (aryl hydrocarbon receptor nuclear translocator-like protein 1), also known as BMAL1, induce the transcription of core clock genes, *Periods* (*Pers*) and *Cryptochromes* (*Crys*) [4,9]. The complex formed by PER and CRY proteins represses CLOCK and BMAL1 transcriptional activity and, in turn, represses their own expression [10]. The CLOCK/BMAL1 complex also controls the expression of genes that form additional regulatory loops. Nuclear receptor transcription factors from the RAR-related orphan receptor (ROR) family and REV-ERBα/β (REV-ERBα: NR1D1; REV-ERBβ: NR1D2) activate and suppress *Bmal1* expression [9,11,12] (Figure 1). In addition to the transcription and translation, the molecular clock is further regulated by post-translational modifications, including phosphorylation, ubiquitination and sumoylation, which control protein stability and the nuclear translocation of the core clock proteins [7].

Disruptions in circadian rhythms are implicated in pathologies such as diabetes, cardiometabolic disorders and cancer [13]. Clinical and preclinical evidence links the circadian clock and tumorigenesis [14]. Circadian disruption by shift-work, jet lag, late night light exposure and late night food binging has been long linked to increased cancer risk [15,16,17,18]. Furthermore, loss of circadian rhythmicity in patients has been associated with poor response to anti-cancer therapies and increased early mortality rates amongst cancer patients [19,20]. Animal models of genetic disruption of clock genes have been strongly associated with different types of cancers such as prostate, breast, colon, liver, pancreas, ovary, and lung cancers [21,22,23,24,25,26,27], supporting the epidemiological evidence. Night shift work involving circadian disruption has been designated as a Group 2A carcinogen by the International Agency for Research on Cancer (IARC), generating interest in the molecular mechanism of circadian dysregulation and tumor development [28].

Mounting evidence supports molecular interconnections between the circadian clock and cancer. Many of the recognized cancer hallmarks such as copious metabolic demands, a favorable inflammatory microenvironment and immune suppression, and resistance to cell death [29] have a circadian component to them. It is thus hypothesized that oncogenic transformation may lead to malfunctioning of the circadian clock, in turn creating a homeostatic imbalance, which facilitates cancer growth and expansion. In this mini review, we discuss a symbiotic relationship between the clocks and cancer as well as its relation with cancer-related metabolic pathways such as IGF-1R/mTOR/Akt signaling that could drive tumorigenesis. We further discuss the perspective of targeting the circadian clock and its rhythms in combination with available anti-cancer therapeutics.

## 2. Method

This mini review is a compilation of recent discoveries in circadian clock, cancer and metabolism. PubMed was searched for the following keywords such as ‘Circadian clock and cancer’, ‘circadian clock and metabolism’, and ‘chronotherapy and cancer’. “Review” filter was applied and the PubMed search showed anywhere between 37–500 search articles, depending on the keywords. We have mainly included recent review articles from 2015 to 2020 that closely match our review topic. Only relevant data that were consistent among different review articles were extracted from these articles. Clinical trials and meta-analyses were excluded from our search since most relevant and important clinical data were extracted from the review articles listed below.

Some of the recent reviews relevant to our review subject that were mainly considered and have been referred to in the Results and Discussion are “Interplay between Circadian Clock and Cancer: New Frontiers for Cancer Treatment” by Sulli et al., “Circadian Clocks and Cancer: Timekeeping Governs Cellular Metabolism” by Verlande et al., “Crosstalk between Metabolism and Circadian Clocks” by Reinke et al., “Metabolic Rivalry: Circadian Homeostasis and Tumorigenesis” by Kinouchi et al., “Cancer and the Circadian Clock” Shafi et al., and “The Cancer Clock Is (Not) Ticking: Links between Circadian Rhythms and Cancer” by Morgan et al. While there are several important pathways involved in tumorigenesis that are discussed in these reviews, this mini review focuses on a brief overview of clock connection with metabolism in cancer risk.

## 3. Results and Discussion

### 3.1. Circadian Disruption in Cancer

Modern lifestyle impacts the clock. Shift work, time-zone traveling and abnormal feeding patterns are major factors that cause circadian resynchronization. Epidemiological studies support the contribution of clock disruption to tumorigenesis. Women working in rotating or night shifts for several years are at ~10–60% increased risk of developing endometrial and breast cancer [30,31,32,33] in a plasma melatonin rhythms dependent [34,35] and independent manner [36,37]. Another study in male subjects has shown high incidences of prostate cancer in night-shift workers with the cancer grade being proportional to the duration of the shift work [17,38]. Flight attendants that have irregular schedules are at increased risk of melanoma, prostate, and breast cancer due to disturbed circadian rhythms [39,40,41].

Animal studies help understand rhythmic disruptions in carcinogenesis. Rewiring of clock rhythms through chronic jetlag or SCN ablation [42,43] are some of the approaches that have been useful to understand the impact of disruption on cancer risk. Wild-type (WT) animals on a chronic jet lag (CJL) paradigm, i.e., repeated 8 h phase advances in the light/dark cycle every 2 days, for several weeks, have demonstrated an increased risk of Glasgow osteosarcoma [43], lymphoma and hepatocellular carcinoma [3,23] incidences. Several other clock mutant mice models such as mutation of *Per2^m/m^*; *Per2^−/−^*, or *Per1^−/−^* [23,44] and double-null *Cry1/2^-/^* model demonstrated enhanced incidence of lymphoma, hepatocellular carcinoma (HCC), kidney, ovarian, intestinal and pancreatic tumors in chronically jetlagged animals [23]. These studies suggest tumor development in both genotypes with lower incidence in WT animals compared to the mutant mouse models. This argues for a tumor-promoting role of clock gene disruption.

Evidence on clock rewiring in cancer is growing. Although the Cancer Genome Atlas (TCGA) database shows a low mutation frequency of clock genes, single nucleotide polymorphisms (SNPs) in clock genes are associated with increased incidences of prostate, lung, colorectal, breast, HCC as well as other tumor types [45,46,47]. Analyses of survival data from cancer patients such as breast cancer, suggests that patients harboring clock gene mutations have an overall lower survival rate compared to patients with no mutations. Furthermore, TCGA and other database analyses show differential expression patterns of core clock genes in tumors compared to the non-tumor controls [48,49]. Importantly, these data need to be interpreted with caution due to oscillation of clock gene expression. For example, clock gene oscillation is impaired in HCC and metastatic melanoma compared to the normal tissues [50,51]. The diurnal oscillation in clock gene expression should be taken into consideration before drawing a conclusion on clock gene expression in the clinical database [52,53].

**Figure 2:**
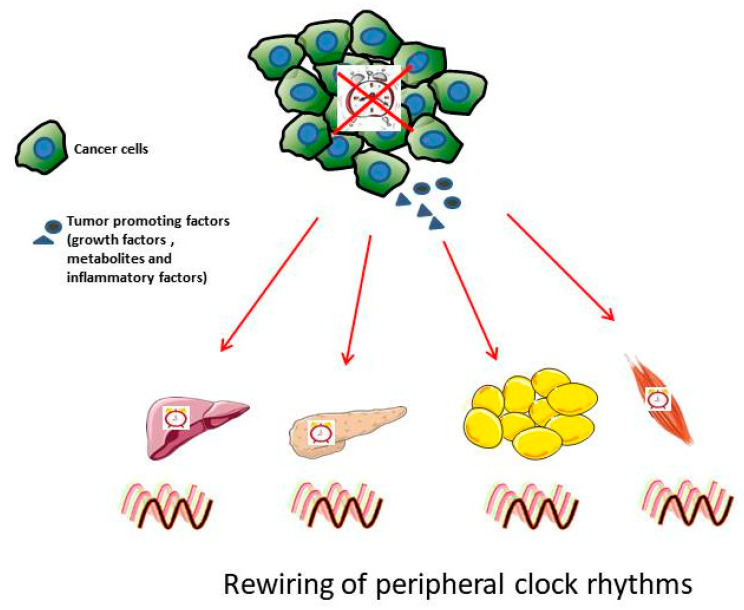
Cancer affects peripheral clock rhythms. Cancerous cells have disrupted circadian rhythms that promote excessive generation of tumor-promoting factors. These tumor-promoting factors, i.e., growth factors such as Transforming growth factor β (TGFβ), and fibroblast growth factor (FGF); inflammatory factors, chemokines and cytokines; metabolic waste byproduct such as lactate are secreted into the blood and travel to the nearby healthy peripheral tissues such as liver, pancreas, adipose tissue and muscle resultingin disturbed rhythmicity in peripheral clocks and its downstream pathways.

Alterations in the expression of clock genes have been observed in animal cancer models (Table 1): *Clock, Rev-erbα*, *Per2*, *RORγ* in the nearby liver tissue of breast-cancer-bearing mice [54], *Per1, Per2,* and *Bmal1* in metastatic melanoma [55], *Rev erb*, *Per2* and *Bmal1* in Glasgow osteosarcoma, and pancreatic adenocarcinoma [56,57], and *Rev-Erbα*, *Per1*, *Per2*, and *Bmal1* in colorectal liver metastases with phase-shift effect observed in nearby healthy kidneys [58]. This suggests that disrupted rhythms are not only observed in the tumor, but also in the nearby distal organs such as liver and kidneys (Figure 2). Further, a study by Masri et al. shows how lung cancer affects rhythmicity in nearby healthy liver at the transcript and metabolites level, demonstrating the impact of cancer on circadian metabolome and clock-controlled genes (CCGs) regulating the downstream signaling pathways [59]. The circadian clock regulates rhythms in the expression of downstream genes known as clock-controlled genes (CCG) [60,61]. It is estimated that about 10–20% of genes in every tissue are CCGs. CCGs regulate multiple signaling pathways such as DNA damage repair, oxidative stress, cell proliferation, and apoptosis. The disruptions of rhythms in tumors impact all of these aforementioned pathways in nearby distal organs. Thus, it is imperative to understand the cross-talk of the core clock genes with key hallmark pathways controlling cancer growth and development. Many studies are addressing the cancer effects on clock in tumors and tumor-free distal organs; however, whether clock disruption in these distal organs also has a role in tumor progression needs to be investigated.

Data on the role of individual circadian clock proteins in tumorigenesis suggests tumor-suppressive as well as -promoting functions of clock proteins. For example, in response to irradiation, *Per2* deficient mice have an increased rate of spontaneous tumor development [44]. Contrastingly, *Clock/Clock* mutants do not develop spontaneous tumors in response to γ-irradiation but do demonstrate overall reduced survival compared to their wild-type counterparts [62]. Further, contrasting results also exist with respect to tumor development in *Cry1*^−/−^;*Cry2*^−/−^ mice with one study reporting γ irradiation induced lymphomas while another study reported no tumor formation in response to γ induced irradiation [23,63]. Additionally, Cry2 deficiency causes accelerated lymphoma development in a c-Myc-dependent manner [64]. *Bmal1*^−/+^ heterozygous mice are prone to lymphoma, HCC and ovarian cancers, with irradiation further increasing the incidence of these and several other cancer types [23]. Clock disruption by jet lag or by genetic ablation (*Per2^m/m^* and *Bmal1^−/−^)* in genetically modified mouse models (GEMMs) such as *K-rasLSL-G12D/+;p53flox/flox* or *K-rasLSL-G12D/+* accelerated lung tumorigenesis [21]. However, contrasting results also exist: *Clock* and *Bmal1* expression is elevated in colorectal cancer and acute myeloid leukemia (AML) and promotes tumor growth in AML [65]. Cry1 and Cry2 deletions suppress tumor development in the p53 null background [66]. Thus, the impact of clock deficiency is cancer-type- and clock-gene-specific. Future studies are required to unravel this complex interplay between cancer, circadian rhythms and clock gene mutations.

### 3.2. Clock and Cancer Metabolism

Various hallmarks of cancer such as DNA damage repair pathways, immune suppression, cell death, cell cycle, tumor-promoting inflammation and cellular metabolism have been proposed to promote tumor growth, and their dissemination has been discussed in detail in a review by Hanahan et al. [29]. The circadian clock control of these cancer hallmarks has been recently covered by Sulli et al. [67]. The circadian clock is a master regulator of metabolism [14,68,69,70,71] and there is evidence of metabolism rewiring in oncogenic transformations [14,72]. The reviews by Brian Altman and Verlande et al. provide details on the complex interplay between cancer-induced disturbed metabolic pathways and peripheral circadian clocks [73,74]. The insulin/IGF1/PI3K/mTOR signaling cascade plays an important role in cancer and metabolism [75,76]. Recently, several groups, including ours, reported on crosstalk between the circadian clock and mTOR pathway. We will discuss how this connection helps to understand metabolic rewiring in cancer.

**Figure 3:**
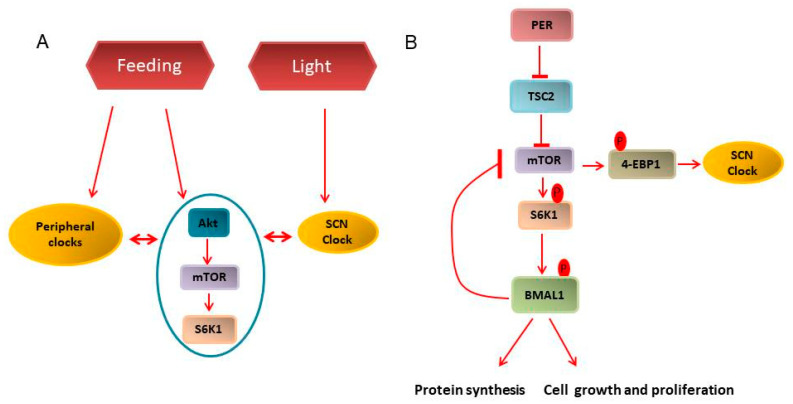
Clock and mTOR pathway interconnection. (**A**) Environmental cues such as light synchronizes the master clock in suprachiasmatic nucleus (SCN) while feeding regimen entrains the peripheral and metabolic clock. Further, peripheral and SCN clocks directly cross-talk with the metabolic clock, i.e., PI3K/Akt/mTOR. (**B**) mTORC1, through 4-EBP1 phosphorylation, regulates the SCN clock. In the periphery, a feedback loop is formed in which mTORC1 regulates BMAL1 activity through S6K1 mediated phosphorylation, while BMAL1 accumulation, in turn, inhibits mTOR activity. Constitutive mTORC1 activation for example, by Tsc2 inhibition, disturbs this feedback loop that could, in turn, drive uncontrolled protein synthesis, cellular growth and proliferation, thus, contributing to cancer development.

The mechanistic target of rapamycin (mTOR) is an important nutrient sensor and orchestrates various downstream anabolic and catabolic processes. Our group has demonstrated that mTOR activity may be under the control of the circadian clock [77,78] (Figure 3A). Indeed, rhythmic oscillations in mTORC1 activities, as indicated by ribosomal protein S6 kinase 1 (S6K1) and 4E-BP1 phosphorylation, is observed in different regions of mouse brain, namely the SCN, arcuate nucleus, hippocampus and frontal cortex, which regulate critical activities, such as, feeding, memory, and learning [79,80]. Rhythmicity in mTORC1 activity has also been shown in different peripheral organs, i.e., retinal photoreceptors, adipocytes, liver, cardiac and skeletal muscles [79]. Interestingly, mTOR and the circadian clock are regulated by common cues, such as food and feeding regimens including calorie restriction (CR) and time-restricted feeding (tRF) [52,81,82,83]. Mechanistically, the circadian clock might regulate mTORC1 signaling through several interconnected pathways (Figure 3B). Insulin/IGF signaling is the main extracellular signal to mTORC1 [84,85], and the circadian clock regulates plasma IGF1 and insulin levels [81,86,87]. Circadian transcriptional factor BMAL1 inhibits mTORC1 activity [77], most likely, through its transcription by PER2, also a clock protein, which directly interacts with mTORC1 during fasting [88]. In turn, mTORC1 regulates the circadian clock. mTORC1 downstream kinase S6K1 phosphorylates BMAL1, which is associated with regulation of translation and protein synthesis [89,90,91]. mTORC1 synchronizes the SCN clock in vivo and affects rhythmicity in peripheral clocks such as liver, and adipocytes [80,92,93,94]. mTOR/4-EBP1-dependent control of vasoactive intestinal polypeptide rhythmicity contributes to the robust oscillation of clock gene expression in SCN [80,92]. Constitutive activation of mTOR in *Tsc2-/-* fibroblasts leads to increased levels of BMAL1, CLOCK and CRY1 [94].

An epistatic relationship exists between clock and mTORC1 not only in healthy cells but in cancer cells also. Rhythms in mTORC1 activity have been demonstrated in mouse renal cell carcinomas, human breast cancer cells and osteosarcoma [95,96]. Knockdown of Bmal1 in different colorectal cancer cell lines leads to activation of the Akt/mTOR pathway, albeit to a different extent, and thus, increased proliferation of cancer cells [97]. Period2 (Per2) overexpression in a cisplatin-treated human lung adenocarcinoma cell line increases apoptosis and reduces cell proliferation by suppressing phosphoinositide 3-kinase (PI3K)/AKT/mTOR [98]. Overexpression of PER1 as well as PER2 in an oral squamous cell carcinoma cell line suppresses tumor growth by increasing autophagy in an PI3K/Akt/mTOR-dependent manner [99,100]. Dysregulation in the tumor suppressor PTEN and increased oxidative stress leads to activation of BMAL1 in an mTOR-dependent manner in cancer cells [101].

### 3.3. Circadian Strategies for Cancer Treatment

**Figure 4:**
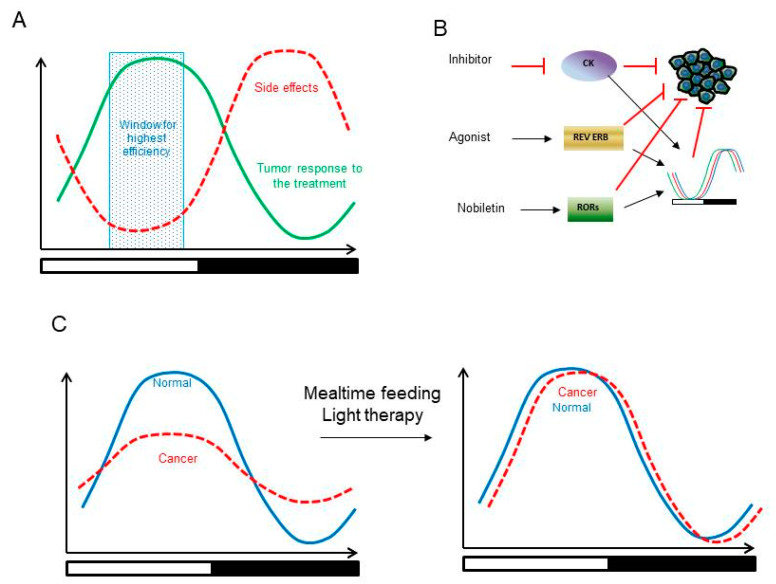
Circadian approaches to cancer treatment. (**A**) Chronotherapy of cancer. Tumor response to treatments, such as chemotherapy or irradiation, is time-of-day dependent. Toxic side effects are also influenced by the rhythms. Knowledge on both rhythms will help to identify a time window for the most efficient treatment. (**B**) Targeting circadian clock proteins directly, RORs or REV-ERBs, or indirectly, CK2, with small molecules impacts the tumor either through the circadian clock and rhythms or through clock-independent tumor suppressor functions of clock proteins. (**C**) Restoring the circadian rhythms using environmental cues, such as time restricted feeding or light exposure, could be beneficial for prevention and treatment of cancer.

Chronotherapy is an experimental approach in cancer treatment. It was proposed that selecting the most appropriate circadian time for the treatment might improve the outcome. Traditional anticancer approaches such as radiation or chemotherapy are associated with severe side effects, which include inflammation, leukopenia, and skin rashes due to damage to the healthy organs caused by off-target effects of the treatment [102,103]. Daily rhythms in cellular pathways such as xenobiotic detoxification or DNA repair may contribute to tumor and healthy tissue response in a time-of-day-dependent manner; therefore, it is possible to use the chronotherapy approach to achieve maximum efficacy [104] (Figure 4). Limited clinical studies are in agreement with the benefits of a chronotherapeutic approach. Radiation chronotherapy suggests improved symptoms with mixed response to overall survival rate in different types of cancers [105]. Chemotherapy dose timing studies have shown lowered adverse events such as inflammation, and leukopenia, i.e., decrease in white blood cell (WBC) count in breast [106], endometrial [107], renal, prostate, cervical, ovarian [108] and colorectal cancer [109,110,111]. For instance, timed dosing of 5-fluorouracil (FU) to colorectal patients reduced mucosal inflammation, while another study demonstrated the effectiveness of a timed infusion of 5-FU in combination with a chemotherapy drug [110,112]. However, due to tumor heterogeneity, variable treatment responses and technical challenges, chronotherapy application in clinical studies is scarce. Several animal studies in circadian clock mutant mice have also demonstrated improved tolerability and efficacy of drugs by optimization of the dose timing [113,114,115]. For example, studies in mice show reduced tumor growth when given cyclin-dependent kinase 4/6 (CDK4/6) drug in a time-dependent manner with high efficacy observed in a morning dosing regimen compared to the night time dosing [56,116]. Another metabolic target, mTOR inhibitor, Everolimus, when administered orally in mice at ZT13 was found to have less severe immunological, biochemical and hematological toxicities compared to dosing at ZT1, further corroborating the need of investigating a chronotherapy approach in large clinical studies [117]. Newer therapeutic options such as immunotherapies targeting immune checkpoint receptors or ligands, CTLA-4, PD-1 and PDL-1 are currently being widely tested in clinical trials for multiple cancer types. However, these are known to show a significant inflammatory response as well as immune response related adverse events [118]. Because immune cell trafficking and the inflammatory pathway are under clock control, applying a chronotherapy approach could help mitigate the associated toxicity issues [119,120,121].

Targeting the clock and its rhythms is a novel direction in chronotherapy of cancer. Modulating the clock with environmental cues such as meal timing and light exposure during night-time is a non-pharmacological approach that is growing in popularity (Figure 4). For instance, overnight fasting of 13 h has been shown to lower breast cancer incidences in women [122]. Further, a study by Marinac et al. presents data from the 2009–2010 National Health and Nutrition Examination Survey (NHANES) on the effect of eating frequency and timing behaviors on breast cancer risk in women. Based on their findings, reducing the food intake in the evening, fasting for long hours at night and consuming frequent meals may help to lower the risk of breast cancer [123]. Feeding time has considerable impact on cancer progression in animal models. For instance, mice with restricted access to food for 6 h during the light phase had reduced cancer growth compared to those freely fed (ad libitum (AL)). This coincides with the rhythmic expression of genes involved in stress response, and cell cycle observed only in restricted feeding mice and not in AL fed animals [57]. Similarly, restricted feeding for 4 or 6 h during the light phase prolonged the overall survival of Glasgow osteosarcoma mouse model possibly due to desynchronized tumor clocks [124]. Timed feeding delayed tumor growth in chronically jetlagged Glasgow osteosarcoma and pancreatic adenocarcinoma models [125]. Circadian disruption by artificial light at night (ALAN) is another emerging risk factor linked to higher risk of breast cancer in women. Mixed data have been reported by several ecological and cohort-based studies worldwide [126,127,128,129,130], suggesting the need for more cohort based studies in larger population. A review article by Richard Stevens gives an excellent overview of studies conducted using different light at night conditions [131]. Studies in animal models such as one conducted in rats exposed to ALAN induced the growth of MCF-7 breast cancer compared to the animals kept under a normal light–dark cycle [132,133]. Another study showed ALAN exposure from lights of different spectral compositions markedly accelerated tumor growth rates in mice with 4T1 breast cancer tumors compared to normal light–dark cycle controls [134,135,136]. Further mechanistic studies are needed to investigate the effect of ALAN exposure on the tumor clock. Light entrains the central clock, in turn, synchronizing the peripheral clock, while food and feeding time directly synchronize peripheral circadian clocks residing in multiple organs. Hence, targeting peripheral clocks along with a non-pharmacological approach such as timed feeding may prove to be an attractive combination due to the following: (1). It will allow for the use of a lower effective dose of pharmacological drug due to an additive effect from timed feeding; (2). targeting the clock in multiple organs will, in turn, target multiple downstream pathways; (3). this would thus aid in reducing the adverse events caused due to either the use of higher effective doses or multiple chemotherapeutic combinations. Thus, restoration of clock rhythms indirectly via environmental factors could aid in lowering the risk of cancer development.

Finally, some of the clock proteins or the clock itself can be targets for pharmacological intervention (Figure 4). Chronomodulating drugs such as dexamethasone, forskalin and melatonin help restore the dampened circadian rhythms in the diseased state. These chronomodulators have been shown to reduce proliferation of a variety of cancer cells by restoration of circadian rhythms [55,137]. Melatonin, a hormone produced in a circadian manner in the pineal gland and retina, is decreased in untreated as well as chemotherapy-treated patients with non-small cell lung cancer (NSCLC). A review by Savvidis et al. discusses in detail the effect of melatonin on cancer [138]. This and a review by Grubisic et al. highlight important articles on how different lighting conditions can impact melatonin levels and how it may aid in lowering cancer risk in shift-workers [138,139]. Melatonin administration at night time reduces lung metastases in a breast cancer mouse model [140,141]. Additionally, a melatonin supplement given at night time significantly lowered the artificial light at night (ALAN)-accelerated breast cancer growth in mice [134,136]. Dose timing is thus critical since melatonin administration at the wrong time of the day can lead to increased tumor growth [142]. For casein kinases, regulators of circadian rhythms, the inhibition of casein kinase 2 (CK2) activity can suppress the human renal cell carcinoma (RCC) cancer cell growth, likely through lengthening of circadian period, highlighting the need for an in depth investigation [143]. Inhibition of Fbxw7, an F-box protein that controls REV ERBα degradation, has been shown to impair pancreatic cancer tumor growth in vivo [144] and treatment with REV ERB agonists suppresses glioblastoma tumors in mice [145]. Activation of RORγ has tumor-suppressive effects on multiple cancer types [146]. Indeed, RORγ agonist, Lyc-55716, is currently in Phase I clinical trials as monotherapy as well as in combination with pembrolizumab (anti PD-1) in patients with advanced solid tumors. Recently, a small molecule, nobiletin, has been shown to have anti-oncogenic effects through restoration of circadian rhythmicity in a cell-type-dependent manner [147,148].

## 4. Conclusions

In several cancer types, down-regulation of the core clock and its downstream clock-controlled proteins have been reported. Here, we highlight the interconnection of the circadian clock with key metabolic pathways in cancer. Disturbances in rhythmicity and connection could lead to tumor initiation and progression. Clinical and preclinical studies demonstrate the potential and further need for investigating the importance of a chronotherapy approach for improving response and dealing with toxicity associated with existing cancer treatment. Overall, chronobiology in cancer research is a vital connection linking tumor metabolism and other oncogenic signaling pathways that warrants further in-depth investigation through large studies to better allow for interpretation of the benefits of chronotherapy.

Shift-work, exposure to light at night, and variable eating habits can have a detrimental effect on health due to misaligned circadian rhythms. A study in mice further suggests exposure to UVR during the morning hours may lower the risk of skin cancer development in humans [149]. It is thus intriguing to speculate that lifestyle modifications such as limited and timely exposure to UVR; physical activity; time-restricted feeding; reducing light at night exposure by installing room-darkening shades or avoiding looking into cell phones before sleeping; and creating favorable lighting conditions for night shift workers may aid in lowering cancer incidences. However, other factors such as genetic and epigenetic alterations of clock genes further add layers of complexity to understanding its impact on tumorigenesis.

Assessing the timing of treatment that coincides with restoration of the circadian rhythmicity of different cellular pathways is challenging [150]. However, advances in newer approaches such as genomics technology, non-invasive real-time circadian monitoring and biomarker testing would open up new avenues for chronotherapy-based approaches [151,152]. For example, disrupted circadian rhythms in daily activity as measured with wrist-actigraphy 3 days before and during the chemotherapy treatment correlates with poor survival in metastatic colorectal cancer patients [153,154,155]. Overall, integration of these different circadian-based strategies is important for cancer prevention and the expansion of novel anti-cancer treatment options targeting clock components.

## Figures and Tables

**Table 1:** Clock gene expression in animal models of different cancer types.

Clock Genes	Animal Cancer Model	Outcome	References
*Rev Erbα, Rorγ* and *Per2*	4T1 Breast Cancer	Downregulation	[54]
*Per1, Per2* and *Bmal1*	B16F10 Melanoma	Downregulation	[55]
*Rev Erbα, Per2* and *Bmal1*	Glasgow osteosarcoma	Downregulation	[56]
*Rev Erbα, Per2* and *Bmal1*	P03 Pancreatic adenocarcinoma	Downregulation	[57]
*Rev-Erbα*, *Per1*, *Per2*, and *Bmal1*	C26 Colorectal liver metastases	Downregulation	[58]

## Data Availability

Not applicable.
